# Advances and Challenges of Bioassembly Strategies in Neurovascular In Vitro Modeling: An Overview of Current Technologies with a Focus on Three-Dimensional Bioprinting

**DOI:** 10.3390/ijms252011000

**Published:** 2024-10-12

**Authors:** Salvatore Mancuso, Aditya Bhalerao, Luca Cucullo

**Affiliations:** 1 Department of Biological and Biomedical Sciences, Oakland University, Rochester, MI 48309, USA; mancuso@oakland.edu (S.M.); abhalerao@oakland.edu (A.B.); 2 Department of Foundational Medical Studies, Oakland University William Beaumont School of Medicine, 586 Pioneer Dr, 460 O’Dowd Hall, Rochester, MI 48309, USA

**Keywords:** in vitro, blood-brain barrier, neurovascular, alternatives, biomaterial, cells, matrix, bioprinting

## Abstract

Bioassembly encompasses various techniques such as bioprinting, microfluidics, organoids, and self-assembly, enabling advances in tissue engineering and regenerative medicine. Advancements in bioassembly technologies have enabled the precise arrangement and integration of various cell types to more closely mimic the complexity functionality of the neurovascular unit (NVU) and that of other biodiverse multicellular tissue structures. In this context, bioprinting offers the ability to deposit cells in a spatially controlled manner, facilitating the construction of interconnected networks. Scaffold-based assembly strategies provide structural support and guidance cues for cell growth, enabling the formation of complex bio-constructs. Self-assembly approaches utilize the inherent properties of cells to drive the spontaneous organization and interaction of neuronal and vascular components. However, recreating the intricate microarchitecture and functional characteristics of a tissue/organ poses additional challenges. Advancements in bioassembly techniques and materials hold great promise for addressing these challenges. The further refinement of bioprinting technologies, such as improved resolution and the incorporation of multiple cell types, can enhance the accuracy and complexity of the biological constructs; however, developing bioinks that support the growth of cells, viability, and functionality while maintaining compatibility with the bioassembly process remains an unmet need in the field, and further advancements in the design of bioactive and biodegradable scaffolds will aid in controlling cell adhesion, differentiation, and vascularization within the engineered tissue. Additionally, integrating advanced imaging and analytical techniques can provide real-time monitoring and characterization of bioassembly, aiding in quality control and optimization. While challenges remain, ongoing research and technological advancements propel the field forward, paving the way for transformative developments in neurovascular research and tissue engineering. This work provides an overview of the advancements, challenges, and future perspectives in bioassembly for fabricating neurovascular constructs with an add-on focus on bioprinting technologies.

## 1. Introduction

Pharmaceutical research and drug development is a significantly costly endeavor [1]. The reason for this dramatic cost increase in research and development is multifactorial. Still, one significant factor is the limited availability of translational relevant in vitro models to facilitate drug development (discovery, screening, and testing) for treating a specific disease. In the pharmaceutical industry, the drug discovery process starts with choosing a disease area and defining the therapeutic need that should be addressed. Once this has been carried out, the process continues to identify the target physiological mechanisms and then screens through many potential drug candidates that could elicit the desired biological activity. Assessing the pharmacokinetic and pharmacodynamic properties of the product, its safety, and its efficacy are key components of the drug development process. A significant part of this process involves the in vivo testing of the most promising drug candidates in preclinical animal models. These are expected to simulate the biological and pathological scenarios with which these compounds are anticipated to interact and/or react in human patients [2]. Unfortunately, most preclinical in vivo studies (≈80%) fail to provide realistic therapeutic outcomes and potential toxicities consistent with corresponding clinical trial results. Indeed, in vivo studies have notable limitations, which include the following: (a) a limited ability to replicate the clinical feature of a specific disease; (b) a limited ability to model the unavoidable inter-patient variability to accurately predict the physiological response to the drug treatment and the potential toxicities and side-effects associated with its use [2,3,4]; (c) issues with the consistent reproducibility of the results; (d) issues with scalability and cost-effectiveness; and (e) ethical controversy.

The ongoing burdens of these unresolved issues are particularly evident in neuropharmacology, where the development of brain-targeting drugs faces the most significant hurdles and yields the poorest results regarding new and/or more effective pharmacological treatments released into the market [5]. To aid the development of more effective neurotherapeutics (as an alternative or a complementary tool to animal testing), a push to develop quasi-physiological and humanizable in-vitro tissue and organ models has ensued and is ramping up. These platforms are now widely adopted in preclinical studies associated with drug discovery for the central nervous system (CNS).

## 2. The Neurovascular Unit

**Figure 1:**
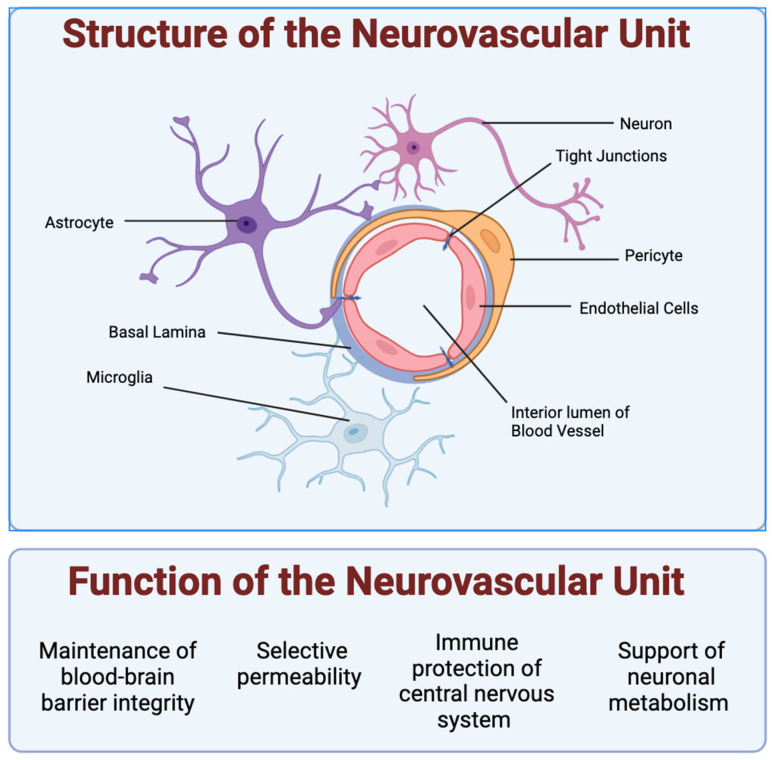
Structure and function of the neurovascular unit: Shown are the 5 types of cells typically studied in the neurovascular unit. Endothelial cells form a lumen for blood to flow. Pericytes encapsulate the endothelial cells and express barrier proteins. Astrocytes and microglia monitor the junction via endfeet projections. Astrocytes help facilitate nutrient exchange to the neurons, while microglia cells act as the resident active immune defense system. The NVU supports neuronal metabolism and the healthy function of neurons in the CNS by enabling the control of oxygen and nutrient levels via vasodilation and vasoconstriction. The NVU cells also make up the blood–brain barrier (BBB), which plays a critical role in maintaining the homeostasis of the brain microenvironment as the gatekeeper of the CNS through strict and selective control of the passage of substances in and out of the brain, including the removal of waste, and protection from potentially harmful substances (endogenous and xenobiotics).

In the neurovascular unit (NVU), each cellular component is vital in sustaining the intricate brain environment (see Figure 1).

### 2.1. Vascular Endothelial Cells

Vascular endothelial cells (ECs) form the core of the BBB, regulating the exchange of substances between the bloodstream and the brain. These cells express tight and adherens junctions that limit paracellular diffusion, ensuring selective permeability. In addition, these specialized brain microvascular endothelial cells express a host of enzymes, including γ-glutamyl transpeptidase, monoamine oxidase, and cytochrome P450, which metabolize and inactivate neuroactive and neurotoxic compounds, peptides, etc. that may reach the brain, as well as efflux transporters (predominantly localized on the luminal plasma membrane) including P-glycoprotein (Pg-p), breast cancer resistance protein (BCRP), and multidrug resistance protein-1 (MRP1), which collectively extrude lipophilic and cationic drugs and xenobiotics from the brain capillary endothelial cells back to the circulation [6,7]. This mechanism is crucial for allowing tight control over the passage of essential nutrients into the brain while blocking potentially harmful substances. However, culturing ECs in static conditions often leads to a loss of these specialized functions and their shear stress sensitivity, owing to the absence of blood flow dynamics in vitro, which can significantly alter their behavior and gene expression.

### 2.2. Pericytes

Pericytes are wrap-around cells on capillaries throughout the body that help maintain blood flow, barrier function, and communication between vessels and the surrounding tissue. Pericytes are integral to the vascular stability and permeability of the NVU and are embedded within the basement membrane of blood vessels [8]. They help maintain vascular integrity, participate in vascular development, and respond to pathological changes such as injury or disease [9]. Pericytes are characterized by their low proliferation rates and dependency on co-culture with other NVU cells like ECs for proper function, complicating their isolation and expansion in culture. Their stem cell-like properties are essential for cellular regeneration within the NVU, highlighting their importance in healthy and diseased states.

### 2.3. Astrocytes

Astrocyte glial cells, named for their star-like shape, provide structural, biochemical, and metabolic support to the CNS. Astrocytes facilitate metabolic exchanges and biochemical signaling, which are crucial for BBB maintenance. Beyond their support roles, astrocytes are situated perfectly between neurons and the endothelium to mediate neurovascular signaling [10,11,12] and control across the BBB. Their endfeet projections extensively cover the vasculature, facilitating and controlling metabolite trafficking and the biochemical signaling necessary for the maintenance of the BBB [13]. Through the secretion of various factors, astrocytes influence the formation of tight junctions in endothelial cells, reinforcing the barrier function against circulating toxins and pathogens. Astrocytes, in cooperation with microglial cells through their mutual communication and cooperation in the NVU, play a crucial role in neuroinflammation and amplifying the neuroimmune responses [14]. Their elaborate branching processes, often poorly replicated in vitro, are vital for their function. The loss of this complex morphology in culture can significantly impair their physiological relevance. Astrocytes also influence the formation of tight junctions in ECs, enhancing the protective functions of the BBB.

### 2.4. Microglia

Microglia are glial cells that act as the primary immune cells in the CNS. They are the resident macrophages of the brain and spinal cord. They are highly sensitive to their environment and play a crucial role in the immune response within the CNS [15].

These cells originate from myeloid progenitor cells and migrate to the brain during early development, where they differentiate and become specialized immune cells. Microglia play a critical role in immune surveillance in the CNS, where they constantly monitor their environment and respond to injury, infection, or disease by activating and releasing inflammatory mediators, phagocytizing pathogens and debris, and helping repair damaged tissue [16]. In addition to their role in the immune response, microglia are also involved in maintaining the brain’s normal functioning by pruning synapses during development, removing excess or damaged neurons, and supporting neuronal health [17]. Dysregulation of microglial function has been implicated in various neurodegenerative diseases [18], including Parkinson’s disease [19], Alzheimer’s disease [20], and multiple sclerosis [21]. In some cases, microglia can contribute to neuroinflammation and neuronal damage. Due to their important roles in health and disease, microglia cells remain the subject of extensive research in neuroscience and neuroimmunology to develop novel therapies for neuroinflammatory and neurodegenerative diseases. In vitro, changes in culture conditions can drastically alter their function and activation state, and they tend to lose viability and characteristic morphology over time.

### 2.5. Neurons

Neurons, while primarily recognized for their role in signal transmission, also actively regulate cerebral blood flow, allowing for the controlled exchange of nutrients and metabolic waste products. Neurons in the NVU transduce signals and can control local cerebral blood flow directly via the release of nitric oxide (NO) and indirectly through interactions with glia cells, where neuron-derived NO regulates glia-mediated vasodilation via prostanoids and epoxyeicosatrienoic acids [22,23]. They directly interact with vascular components to facilitate increases in blood flow in response to active metabolic demands, a phenomenon known as functional hyperemia. This ability of neurons to regulate blood flow underscores their critical role in ensuring that active brain regions receive adequate blood supply in real time [24]. However, from an in vitro experimental point of view, neurons present unique challenges due to their non-proliferative nature and dependence on specific growth factors and cellular coupling for their survival and functional viability.

Developing an in vitro NVU model that encompasses and reproduces the functional association of neurons in the brain and the BBB is crucial not only for advancing our understanding of regulatory brain functions and how disease conditions impact them but also for facilitating the development of novel and more effective therapeutic strategies to treat neurological disorders. Integrating biomaterials into these models to establish the structural and environmental cues needed to promote realistic cell interactions (and the corresponding functional responses) is paramount to developing more accurate and reliable in vitro platforms for basic, translational, and pharmacological studies.

## 3. Bioassembly

Bioassembly is an emerging biofabrication technology process using living cells or composite biosynthetic materials (cells embedded in a synthetic matrix) to create relatively complex three-dimensional biological structures with a pre-set spatial organization [25]. This can be achieved via cell-driven self-assembly or bottom-up fabrication technologies and biomaterial assembly (e.g., bioprinting) [26]. It has diverse applications in regenerative medicine, tissue engineering, drug discovery, and basic research [27]. Cell–cell interactions play a vital role in bioassembly, allowing for the precise organization of cellular behavior and function within a three-dimensional context, mimicking natural tissues [28]. Techniques like self-assembly [29], bioprinting [30], and cell sheet engineering [31] enable the formation of complex structures with high levels of cellular organization and function. Cell–cell interactions create dynamic cellular environments that mimic those found in natural tissues and promote cellular differentiation, proliferation, and tissue-specific function.

In the NVU context, bioassembly can be used to generate in vitro models mimicking the complex cellular interactions and functions of the brain’s microvasculature [32]. Microfluidic devices and organoid cultures are two approaches to bioassembly in the NVU [33]. Microfluidic devices allow the establishment of a quasi-physiological microvascular environment outside a living organism that can closely mimic (functionally and behaviorally) that in vivo. These platforms allow for precise control and manipulation of many biological and physical variables to affect and modulate the behavior and functions of cells [34,35]. Organoids are three-dimensional structures generated from stem cells or other cell types that can mimic, to some extent, the structure and function of specific tissues, including the brain [36].

### 3.1. Role of Cell–Cell Interactions in Bioassembly

Cell–cell interactions are critical in bioassembly to create complex three-dimensional structures mimicking natural tissues [26]. The controlled assembly of cells and biomaterials allows for the precise organization of multicellular structures within a three-dimensional (3D) context [30] to promote cellular differentiation, proliferation, and the development of tissue-specific function and physiological responses similar to those observed in situ, in the corresponding tissue/organ [37].

One approach to achieving cell–cell interactions in bioassembly is using cell aggregates or spheroids [32]. These are clusters of cells formed through self-assembly or aggregation, allowing for the development of complex 3D structures with high cellular organization and function levels. Another approach is using cell sheet engineering, which involves the creation of layered structures of cells grown on temperature-responsive polymer surfaces [38]. These layered structures can be stacked and manipulated to create more complex yet precisely organized three-dimensional structures.

Another bioassembly method in the NVU context involves using microfluidic culture devices [39,40]. These in vitro platforms allow the development of in vivo-like microvascular structures capable of reproducing not only the three-dimensional and multicellular environment (including endothelial cells, pericytes, and astrocytes) of blood vessels but also a wide range of hemodynamic cues that these vessels are exposed to under normal and pathological conditions [34]. These platforms have practical usability, not only in basic research when studying interactions between the various cell types that form blood vessels under normal and pathological conditions in a highly controllable environment, but also for a wide range of pharmacological and toxicological studies [33,40,41,42].

A biocompatible multicellular environment organized in a physiologically relevant three-dimensional structure, where cells can be exposed to in vivo-like biological (from the surrounding multicellular milieu) and/or physical cues (environment-/function-dependent), can promote in situ-like cellular re-organization, differentiation, and the expression of functional responses mimicking that of the corresponding tissues in vivo [26,33]. This allows for the studying of natural cell–cell interactions and functional responses, including mechanisms underlying tissue development and disease progression, as well as toxicological and pharmacological responses to endogenous substances and xenobiotics, including putative therapeutic treatments.

### 3.2. Organoid Cultures and Bioassembly

**Figure 2:**
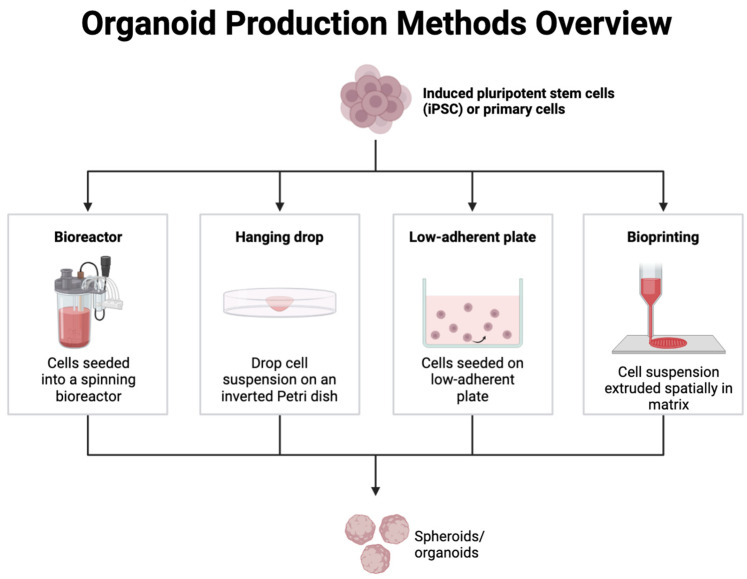
Schematic overview of current methods for organoid production. From left to right: bioreactors, hanging drop method, low-adherent plate, and the most recent, bioprinting.

Another approach to bioassembly in the NVU is using organoid cultures [36]. Brain organoids can recapitulate cellular and architectural aspects of certain brain regions [43,44,45]. They comprise self-organized three-dimensional neural aggregates derived from pluripotent stem cells or organ progenitors. Through cell sorting and spatially restricted lineage commitment, these aggregates can undergo a morphogenic process, resulting in the development of microscale tissue cytoarchitecture and cell phenotypic diversity that are not otherwise achievable [46]. For more detailed information, we point the reader towards more specific literature on the topic [47,48]. Brain organoids can be used to model the cellular interactions and functions of the NVU, including the formation and maintenance of the BBB and the interactions between neurons and glial cells [45,49]. Brain organoids can form vascular networks [50] that resemble the in vivo microvasculature of the brain, allowing for the study of BBB function and the interactions between cells in the NVU. Organoids also offer the potential for high-throughput screening of drugs and other therapies [51]. Multiple organoids can be generated simultaneously, and their response to different treatments can be measured in a somewhat reproducible and controlled manner, providing a platform for basic drug discovery [49,52,53,54]. Different methods are currently available to produce organoids in vitro (see also Figure 2) as briefly described below, including:

- Bioreactors: In a bioreactor, the culture medium with cells is mixed continuously to ensure an even distribution of nutrients and oxygen while removing waste products, thus providing an optimal controlled environment for cell growth and differentiation to cultivate organoids [55].
- Hanging drop method: This air–liquid interface technique relies on the accumulation of cells at the liquid–air interface to form spheroids. Small droplets of cell suspension are placed on the lid of a petri dish, which is then inverted. Surface tension and gravity cause the cells in each droplet to aggregate at the bottom of the drop, where they self-organize into three-dimensional structures [56].
- Low-adherent plates: These plates are coated with materials that prevent cells from attaching to the surface, encouraging them to aggregate into spheroids or organoids. As the cells cannot adhere to the plate, they naturally cluster together, mimicking the three-dimensional architecture of tissues [56].
- Bioprinting: This technique can combine cell suspensions, biomaterials, and active molecules to print a 3D structure layer-by-layer. Bioprinting methods such as extrusion-based, inkjet-based, and laser-assisted bioprinting have been used to produce organoids [57].

Neural organoids have become an invaluable approach in modeling features of human brain development that are poorly reflected in animal models and provide a promising tool for modeling the complex cellular interactions and functions of the NVU [49,52,53], in order to unravel neurobiology features of neural development and diseases. However, despite their promising features predominantly related to the development and ability to study neuron–neuron or neuron–glial interaction, the broad utility of organoids is hampered by several limitations. Organoids often lack key specialized cell types and may not be able to recapitulate the complexity of native organs, including functional vascular systems, microbiome, etc. In addition, the limited cell maturation, lack of high-fidelity cell types, and the lack of consistent cellular organization hinder their reliability and robust experimental readouts in certain applications [58]. Although vascularized organoids (vOrganoids) further increase the translational relevance of this model, integrating both vascular and neuronal physiology within the system [59], the inability to perfuse the vascular structures of organoids in an active and controlled fashion aside from simple diffusion (since the vasculature in these vOrganoids exhibits only structural features without complete functionality [60]) significantly reduce their usability for a wide range of pharmacological as well as pathological studies. This issue has been partially solved with the recent development of vascularized organoid-on-a-chip (VOoC), where the vascular endothelium assembles on pre-patterned vessel lumens, allowing for controlled fluid perfusion of these vessels [61]. The VOoC vasculature can then be characterized by combining high-resolution microscopic imaging coupled with tissue optical clearing (TOC) techniques to allow for their 3D visualization [61]. However, conventional organoid culture techniques remain quite time-consuming and not fully scalable with a low level of reproducibility because of organoid spatial self-organization, which remains quite unpredictable. To address some of these scalability/reproducibility problems, there has been a recent push toward developing 3D bioprinting technology for organoids [57]. Generally, the bioprinting of organoids, including kidney, liver, heart, intestinal tract, and even tumor models, has been modestly successful [62]. Still, this technology has recently found an application in the bioprinting of brain tissue organoids with some partial success, given the higher level of complexity of the tissues [63,64]. Significant technological advancements in the field will, however, be necessary to preserve the organoid structural and cellular complexities while improving the scalability and reproducibility of the system.

### 3.3. Microfluidic Platforms and Bioassembly

While organoids enable the reproduction and study of brain tissue architectures and physiological functions, other groups have focused on the cerebrovascular facet of the brain, particularly the BBB, and the development of experimental BBB in vitro models suitable for developing novel CNS therapeutics that require BBB crossing, as well as brain disorders linked (directly or indirectly) to altered neurovascular functions.

**Figure 3:**
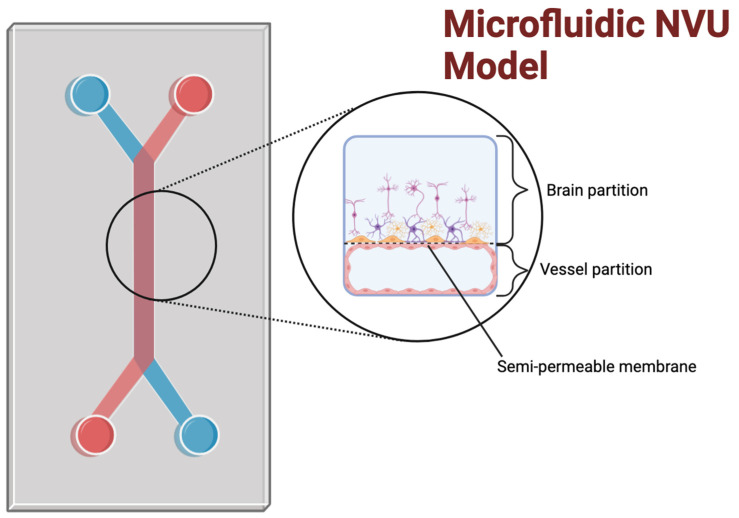
An example of a microfluidic model of the neurovascular unit: Microfluidic models typically incorporate a two-chamber design; the upper channel is used for the brain partition, and the smaller, lower channel is used for the vascular partition, allowing the two distinct microenvironments to be spatially separated. A semi-permeable membrane separates the upper brain partition from the lower vessel partition. This allows for the exchanging of biomolecules and cell–cell interactions within and across the compartments. Flow induction along each channel can also be varied independently to simulate different dynamic conditions. This is only one example of the possible architectures of microfluidic chip designs. Other patterns for different use cases and cell volumes are possible.

In this respect, microfluidic cell culture devices (see Figure 3) enable cell cultures to be exposed to fluid flow dynamic conditions and have been used to develop in vitro models of the NVU through bioassembly [40,65]. These devices use the precise control of fluid flow, cell seeding, and other environmental factors that can influence cellular behavior and function to create an environment that more closely resembles the in vivo microvasculature of the brain [34]. Along with co-culture, shear stress is an important factor for accurately modeling the cell phenotype of endothelial cells [66,67]. Microfluidic devices allow for control over shear stress, an important differentiation factor for maintaining the morphology of endothelial cells [68], which also promotes bioassembly and provides control over other aspects of the model’s architecture, such as shape and compartmentalization. The advantage of multi-compartment microfluidic chips for NVU fabrication is that, with separate channels, different environmental, mechanical, and multicellular biological cues [69] can be provided to promote cell/tissue differentiation as well as for experimental testing. Each cell type can be isolated in its appropriate channel to model the original vascular tissue’s spatial cellular distribution and topographic connectivity. Within their corresponding compartments, cells can be exposed to culture media most closely resembling what the cells experience in situ (e.g., vascular vs. parenchymal fluids) as well as differential physical factors (blood flow-dependent shear stress on the vascular side vs. quasi-static conditions in the brain side). The use of semi-permeable membranes between the compartment facilitates cell–cell interactions by allowing these cells to be exposed to each other secreted biological factors as well as physical interactions between juxtaposed cells (e.g., astrocytes endfeet connecting to the endothelial basal membrane through the micropores within the separating membrane; see also Figure 3). The membrane also serves as scaffolding support for cell adhesion and growth and can be differentially/specifically coated for the type of cells used within each compartment.

Microfluidic models of the NVU have been created using induced pluripotent stem cell (iPSC)-derived brain microvascular endothelial cells (BMECs), juxtaposed with primary human pericytes and astrocytes in a co-culture to enable BBB-specific characteristics [34,70,71,72].

Incorporating endothelial cells, pericytes, and astrocytes in microfluidic devices is crucial for modeling the NVU [73], as these cell types play critical roles in forming and maintaining the BBB [74]. Microfluidic devices can be designed to incorporate these cell types in a perfusable vascular network, enabling the study of their interactions and functions [33]. Additionally, reproducing physiologically relevant fluid flow patterns of the brain microcapillaries allows us to investigate the effects of flow on BBB function and mimic cerebrovascular pathological conditions that can hamper the NVU [40].

In addition to fluid flow, these microfluidic platforms allow control over other environmental factors, such as oxygen tension, pH, the concentration of growth factors, and other signaling molecules. These factors can influence cellular behavior and function [33], and by controlling them in a precise and reproducible manner, microfluidic devices can create an artificial microenvironment that is closely bioequivalent to their in situ counterpart [70,73,75]

### 3.4. Bioprinting and Bioassembly

Bioprinting is an emerging technology that can potentially create in vitro NVU models through bioassembly [76]. Bioprinting involves the use of 3D printers that concurrently deposit both living cells and biomaterials layer-by-layer to create three-dimensional structures; this approach leverages several advantages afforded by bioprinters over traditional culture, including precise control over cell and scaffold placements, to create 3D biostructures [77,78,79]. Bioprinters could potentially allow for the development of multicellular biological constructs via the controlled simultaneous deposition of cells and biomaterials with micron-level precision to mimic the spatial organization and cellular distribution of the corresponding tissue in situ. By contrast, two-dimensional (2D)/3D matrigel culture systems can also be used to develop vascular tubes in vitro via a self-governing bioassembly process enabled by properly timed and distributed growth factors. Still, the spatial characteristics cannot be as precisely controlled, nor does it allow for the formation of perfusable luminal space within the vessel, thus limiting the use of this platform primarily to studies related to angiogenesis [80].

**Figure 4:**
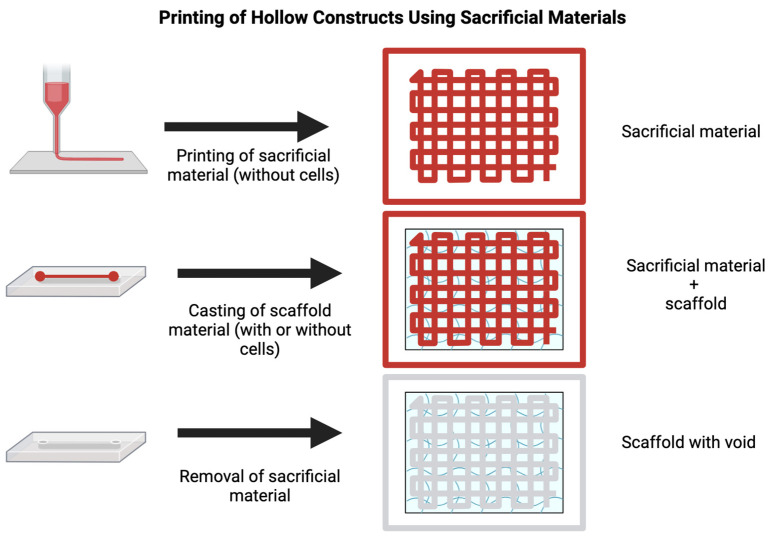
Printing of hollow constructs using sacrificial materials: The fabrication of constructs with hollow voids is possible using a sacrificial material such as pluronic. First, the sacrificial material is printed in the desired shape of the final void. Then, the scaffold material is cast or printed atop and around the sacrificial material. Finally, the sacrificial material is removed, following the solidification of the scaffold material. This leaves a construct with a void in the negative shape of the architecture printed using the sacrificial material. This is useful for fabricating micro- and millifluidic devices and emulating larger vascular units.

One aspect of bioprinting for creating vascular models is the incorporation of multiple cell types [76]. By printing multiple cell types together, researchers can mimic the complex cellular interactions that occur in the in vivo microvasculature of the brain. This is accomplished by encapsulating cells in their scaffold material and printing each scaffold separately in the same construct. This process offers an advantage over traditional co-culture systems, as it can more accurately reconstruct the spatial feature and cellular distribution of native microenvironment in vivo. As such, bioprinting could provide a valuable tool for creating specialized vascular structures [77,81], such as the BBB [76,82]. For example, Lee and co-workers developed a 3D bioprinting method to create a perfused vascular channel layered with a confluent EC monolayer [83]. The cells were exposed to physiological shear stress, remaining viable for 2 weeks and exhibiting barrier properties, including the expression of the endothelial adhesion molecule VE-Cadherine (which controls cellular junctions) and restricted permeability to plasma protein and dextran molecules. In this case, the investigators used a multi-step layer-by-layer process, where a sacrificial material is first printed (gelatine) to form a lumen. This sacrificial material will determine the shape of the lumen, acting as a negative for the void within the lumen. A second material containing the cells is then printed or cast on top of the negative (collagen). After the second material has solidified, the sacrificial material is removed, leaving a void representing the vessel’s lumen. Another recent strategy is to print the blood vessel structure directly utilizing co-axial bioprinting technology, which enables the fabrication of concentric cell-material layers [84]. In this case, the core bioink becomes embedded within another bioink, potentially containing different cell types to recreate a more physiologically relevant vascular tissue (see also Figure 4).

Bioprinting allows for the on-demand creation of many different lumen architectures; however, several technological constraints currently temper the creation of more complex neurovascular systems. For example, bioprinters have limited resolution capabilities, particularly when printing intricate and fine structures. Microvessels have complex geometries, including narrow diameters, intricate branching patterns, and varying wall thicknesses. Achieving high-resolution printing to replicate these features accurately is quite difficult. Creating microvessels often requires the deposition of multiple layers of bioink to form the vessel walls. The alignment and integration of these layers during the printing process can be challenging, as misalignment or poor adhesion between layers can compromise the integrity and functionality of the microvessel. Microvessels require support structures during printing to maintain their shape and prevent collapse. However, incorporating these support structures and allowing their removal post-printing remains a daunting challenge which can also compromise the viability of the cells.

Furthermore, if not properly degraded, these support structures can hinder cell infiltration and interaction with the surrounding microenvironment, thus forming a functional microvascular network. Bioprinted microvessels need to mature and develop functionality to resemble native vessels. This includes endothelial cell alignment, tight junction formation, and the establishment of perfusable lumens. Achieving these complex physiological features in bioprinted microvessels is still an ongoing challenge. Nevertheless, a 3D-bioprinted NVU model encompassing human primary astrocytes, pericytes, brain microvascular endothelial cells, and patient-derived glioblastoma cells was recently developed to reproduce glioblastoma tumor growth and was used to test several chemotherapeutics and anti-cancer drugs and assess the pharmacological relevance of the model for high-throughput screening [85]. Furthermore, Wang and colleagues [86] have fabricated a 3D hollow coaxial neurovascular model showing tight junction expression between the adjacent endothelial layers and selective permeability, while the astrocytes surrounding the endothelial layer were found to directly interact with them via astrocytic endfeet reaching out to the endothelial layer from the outside (brain side) of the vessel.

Another critical factor that needs to be considered during bioprinting is the bioink or bioinks used in the process. In particular, the mechanical, chemical, rheological, and biological properties of the bioink (s) determine factors such as biocompatibility, biomimicry, biodegradability, non-immunogenicity, the mechanical strength and robustness of the construct to maintain the shape fidelity and resolution of the scaffold post-printing, its suitability for chemical modification, and its production scalability with minimum batch-to-batch variability; they also enable suitable cell viability, adhesion, migration, and proliferation. Moreover, the bioink formulations are often determined by the cell type they are intended to be used with to accommodate the necessary nutrients, growth factors, and physical characteristics that maintain cell viability, allowing them to thrive and function properly [87,88,89,90]. The following section provides an overview of the bioinks currently available for 3D bioprinting.

## 4. Bioinks for Bioprinting

### 4.1. Naturally Occurring Bioinks

3D bioprinters share some characteristics with their thermoplastic extrusion 3D printers, which are common in library maker labs and in industry. Rather than extruding a plastic with differential flow characteristics at different temperatures, 3D Bioprinters (or “Bioprinters”) use bioinks, which are low-viscosity suspensions of biomaterial, with or without viable cells, that can be deposited over biologically compatible support, including culture dishes, polymer constructs, or hydrogels, to create quasi-physiological artificial cellular microenvironments [91]. These bioink materials can contain various bioactive molecules and mimic the extracellular matrix (ECM) environment to support cell adhesion, proliferation, differentiation, and viability after printing. Bioinks must have stringent characteristics to be compatible with the intended biological load and supporting functions. These include printing at temperatures within the physiological ranges, forming mild ionic or covalent bonds between polymer chains (mild cross-linking), or forming a three-dimensional gel network (gelation) through chemical or physical cross-linking. Furthermore, bioinks must include non-toxic bioactive components that the cells can alter post-print [92,93].

A wide variety of natural-based materials [94] are used for bioinks, with a current ongoing shift toward synthetic material such as tunable and biodegradable poly (α, L-amino acids)-based bioinks [95]. The major advantage of naturally derived materials, such as alginate, chitosan, collagen, gelatin, fibrin, hyaluronic acid, etc., is their intrinsic bioactivity, good biocompatibility, and biodegradation properties. However, they have modest mechanical properties, which translate into difficulties in printing, the formation of less rigid tissue structures, and reduced mechanical support for the cells in the tissue. The opposite is true for synthetic materials with much better mechanical properties but lacking in biocompatibility, degradability, and potential toxicities. Thus, the selection of a bioink depends on multiple factors, encompassing its biocompatibility, stability, printability, and biodegradability. Furthermore, the viscosity of the bioink plays a major role in the survival of the cells during the printing process since it affects the shear stress to which the cells are subjected when the biomaterial exits the nozzle of the bioprinter [96].

Below is a brief overview of the most common type of bioinks currently in use; more detailed descriptions of their properties and characteristics can be found elsewhere [93,97,98,99].

#### 4.1.1. Carbohydrate-Based Bioinks

Agarose is a polysaccharide bioink derived from red seaweed that has high stability levels and low cytotoxicity levels. Agarose is mainly used in tissue engineering applications, due to its biocompatibility and thermo-reversible gelation and physiochemical properties, which make this biomaterial ideal for supporting cell growth mechanisms [100] and in the construction of microchannels [101]. However, agarose is not degradable and has poor cell adhesion properties. While it offers structural support for tissue development, it may require modifications or a combination with other materials to enhance cell interactions and biodegradability to meet specific application requirements. Agarose has good structural characteristics and biocompatibility, and with some modifications to promote cell adhesion, may be a promising candidate for neurovascular tissue engineering.

#### 4.1.2. Alginate

Alginate is a biopolymer derived from brown algae. Alginate hydrogels are highly biocompatible and biologically inert; their viscosity and mechanical properties can be altered, and the gelation process is quite rapid [102]. Furthermore, alginate hydrogels can be modified by including mammalian cell-interactive domains to improve their biological activity and versatility, thus extending the number of applications and procedures for use. Furthermore, although alginate is slowly degraded and has poor cell adhesion properties, it can, in combination with other components such as gelatin, the product of denatured collagen which possesses cell-adhesive properties, also be used as a thermosensitive bioink [103], and be used to modulate the physicochemical properties and create hybrid alginate hydrogels for different cell culture requirements, including enhanced cell interactions and controlled matrix degradation [104]. Alginate may also be useful in constructing a neurovascular model, for reasons similar to those for agarose.

#### 4.1.3. Chitosan

Chitosan is a polysaccharide with a high degree of structural similarity to natural glycosaminoglycans (GAGs), and it is derived from the exoskeleton of shellfish such as shrimp or through fungal fermentation. As a bioink, chitosan has been investigated for various tissue engineering applications and drug delivery due to its high biocompatibility and biodegradability, low immunogenicity, and cationic nature [105]. Chitosan has some antibacterial properties, which can be beneficial in wound-healing applications. Furthermore, chitosan can be mixed with other bioinks to modify its physicochemical properties [106]. It can also be functionalized with methacrylic anhydride so that the resulting polymer can be photo-crosslinked [107]. Chitosan–catechol has also been developed as an adhesive polymer bioink capable of forming 3D constructs in normal culture media via rapid complexation with serum proteins [108]. Chitosan-based bioinks have some downsides. Chitosan gelation is generally slow, and, in its unmodified form, it dissolves in acidic pH due to strong intermolecular hydrogen bonds. Chitosan also requires a very specific pH for cross-linking, making it less suitable for neurovascular modeling.

#### 4.1.4. Protein-Based Bioinks

Fibrous proteins, including collagen, fibrin, elastin, etc., are characterized by highly repetitive amino acid sequences that provide mechanical and architectural functions in nature.

Collagen is the primary structural protein in the skin and other connective tissues. It is highly biologically relevant and crucial to maintaining tissue integrity and function. Twenty-nine types of collagens have been identified so far, although collagen type 1 is by far the most common (>90% of the total), with types 2 to 4 following far behind [109]. Therefore, collagen type 1 is the most widely used in the formulation of bioink for tissue engineering and bioprinting, due to its biocompatibility and ability to support cell adhesion, proliferation, and differentiation. Most of the collagen bioinks available in the market are based on soluble fiber-free collagen. Thus, fibrillogenesis of this collagen type is required at some point in the printing process. Indeed, under physiological conditions (neutral pH and 37 °C), collagen type 1 starts to self-organize into fibrils with good tensile strength and flexibility. These fibrils can then be cross-linked to support mechanical stimulation [110]. However, low collagen concentrations are generally used in the formulation of collagen-based bioinks to reduce the density of the bioink and the pressures needed for its pneumatic extrusion, which could otherwise compromise cell viability. The resulting effect is a bioink with significantly reduced mechanical properties [111]. Furthermore, collagen is highly sensitive to pH and acid conditions, and the hydrodynamic properties change due to the solution used to solubilize it [112]. However, recently, the use of native, non-soluble, fibrillar collagen inks has been shown to provide high cellular biocompatibility and remarkable cellular viability while maintaining their mechanical properties [113]. Collagen is potentially a highly useful material for neurovascular tissue engineering; however, the difficulty of printing with collagen-based bioinks negatively affects the serial reproducibility of the product.

#### 4.1.5. Fibrin

Fibrin is an insoluble protein that plays a vital role in blood clotting [114]. It has high biological relevance and is involved in wound healing and tissue repair. Fibrin derives from the polymerization of fibrinogen by the action of the serine protease thrombin [115]. Because of its biocompatibility, biodegradability, and tunable mechanical properties, fibrin can be used as a bioink in bioprinting applications. However, its printability is often limited due to its rapid gelation kinetics, making it challenging to precisely control the deposition and patterning during bioprinting. Furthermore, the high viscosity of fibrin in its cross-linked form hinders ink extrusion, and fibrin scaffolds have, per se, low mechanical stability and undergo rapid degradation [116]. Combining fibrinogen with other bioinks can improve its printability and stability [117].

#### 4.1.6. Elastin

Elastin is a highly elastic component of the extracellular matrix (ECM) in the connective tissue of various organs, such as the lung, bladder, arteries, elastic cartilage, and skin [118]. Elastin is a cross-linked network of tropoelastin molecules consisting of alternating hydrophobic and hydrophilic domains. The enzyme lysyl oxidase cross-links the tropoelastin molecules via their lysine residues with desmosine and isodesmosine units. Bioactive elastin-based hydrogels have been recently shown to promote immune cell recruitment and angiogenesis, thus providing a viable tool for wound healing and tissue regeneration [119].

### 4.2. Synthetic Bioinks

#### 4.2.1. PCL, PLA, and PLGA

PCL (polycaprolactone), PLA (polylactic acid), and PLGA (poly (lactic-co-glycolic acid)) are biodegradable thermoplastic polymers and/or copolymers commonly used in tissue engineering and bioprinting. In particular, integrating biodegradable polymers and stem cells with bioprinting techniques has provided significant opportunities for their use in tissue repair, organ transplantation, and energy metabolism [120]. They exhibit high strength and rigidity, making them suitable for constructing scaffolds and implants in regenerative medicine applications. These polymers can provide mechanical support and maintain structural integrity during tissue regeneration. However, PCL, PLA, and PLGA have relatively low cell adhesion and proliferation properties. To address this limitation, surface modifications or incorporation of bioactive molecules may be necessary to enhance cell–material interactions and promote cellular responses within the constructs. The function of the polyester frame has been recently shown that it can be expanded to a composite matrix with smart stimuli-responsive behavior [121]. A biodegradable polyurethane matrix incorporating PCL, polylactide, and poly (3-hydroxybutyrate) (PHB) was recently shown to promote cell proliferation and neural differentiation of neural stem cells. Furthermore, combining chitosan with the polyurethane matrix further improved the bioactive features of the material while demonstrating self-healing properties and ease of bioprinting without the need for a post-crosslinking process of NSCs [122]. PCL may provide a useful solution for developing in vitro structures where larger-scale durability is crucial, such as fluidic chips or large-scale tissue models.

#### 4.2.2. Pluronic

Pluronic (Pluronic F127) is a poly (ethylene oxide) and poly (propylene oxide) block copolymer [123]. It is often used as a sacrificial bioink for temporary support or to create channels, vessels, or vasculature for 3D bioprinting applications [124,125,126]. Pluronic can be printed at room temperature, offering convenience and flexibility in bioprinting [127]. It is also a shear-thinning material, meaning it reduces viscosity under shear stress, allowing for easy extrusion and precise deposition during printing. However, pluronic is not suitable for long-term cell culture. While it provides short-term support and structural integrity, it lacks the necessary bioactive cues and mechanical properties required for sustained cell growth and functionality. Therefore, additional modifications or combinations with other bioinks may be required to create a suitable environment for long-term cell culture in bioprinted constructs. For example, nanostructured pluronic hydrogels, obtained by mixing acrylate with unmodified pluronic F127, demonstrated improved cell viability [128]. Using coaxial nozzles, a combination of unmodified pluronic F127 with F127-bisurethane hydrogels has been used to fabricate hydrogel tubes via the coextrusion of the two shear-thinning materials. These tubes were further functionalized with collagen I, enabling the luminal adhesion of human umbilical vein endothelial cells [129].

### 4.3. Other Bioink Types

#### 4.3.1. Graphene

Graphene is a carbon-based material. It consists of a planar sheet of graphite that is one atom thick. Its unique properties make it valuable in various fields, including flexibility and exceptional electrical conductivity. However, graphene has low biological relevance in bioink formulation and bioprinting applications. It lacks inherent bioactivity and does not directly contribute to cellular processes or tissue development. To utilize graphene in bioprinting, the material is often functionalized or combined with other bioactive materials to enhance its interaction with cells and tissues.

Nonetheless, it is used for applications requiring mechanical and conductive properties. For example, human mesenchymal stem cells (hMSCs)-laden graphene oxide (GO)/alginate/gelatin composite bioink was recently used to form 3D bone-mimicking scaffolds using a 3D bioprinting technique [130]. In addition, a combination of 3D printable GO using Alg and gelatin (Gel) as the basis of a bioink was successfully used to support the growth of adipose tissue-derived stem cells (ADSCs) [131]. More recently, GO-based hydrogel mixed with a paste-like decellularized pancreatic extracellular matrix was shown to possess enhanced mechanical properties, including elasticity, better print resolution, fiber stability, higher water absorbability, and biodegradation, making this material suitable for the biofabrication of tissue models with a vascular system [132]. As such, graphite shows promise when mixed with other biomaterials for neurovascular modeling. Furthermore, its electrical conductivity makes it a potential choice for developing in vitro models when measuring electrical conductance (such as trans-endothelial electrical resistance—TEER or neuronal activity) is desirable.

#### 4.3.2. Hyaluronic Acid

Hyaluronic acid is a non-sulfated glycosaminoglycan that is naturally distributed widely throughout the body’s connective, epithelial, and neural tissues. When used as a bioink, HA exhibits fast gelation properties, allowing for the rapid formation of a gel-like structure. HA is known to promote cell proliferation, making it suitable for tissue engineering and regenerative medicine applications. In addition, this biocompatible, biodegradable, and bioresorbable material allows for the easy diffusion of nutrients. However, one limitation of HA as a bioink is its poor stability and mechanical properties [133], as it can degrade relatively quickly over time. Therefore, hyaluronic acid is generally used with other components, including fibrin and gelatin or other materials (such as hydroxyethyl-methacrylate-derivatized dextran) [133] and, further, with additional enzymatic and photo-crosslinking strategies to enhance its stability and prolong its functionality in bioprinting applications. However, using hyaluronic acid risks damaging any cells embedded during the ultra-violet curing process, so direct bioprinting is not as viable.

The addition of synthetic nanostructured materials of a metallic, polymeric, or ceramic nature is also a viable strategy to enhance the overall characteristics of protein-driven bioinks [134,135,136]. Other components that can also be added to protein-based bioinks include dextran, cellulose, gellan gum, polyethylene glycol (PEG) [98], as well as synthetic materials such as pluronic F127 (also known as Poloxamer 407) [137] and PCL (a biodegradable thermoplastic often used for bioprinting of hard tissues) [138]. Bioink materials and their properties can be found in Table 1.

## 5. Bioprinting

### 5.1. Direct Bioprinting

**Figure 5:**
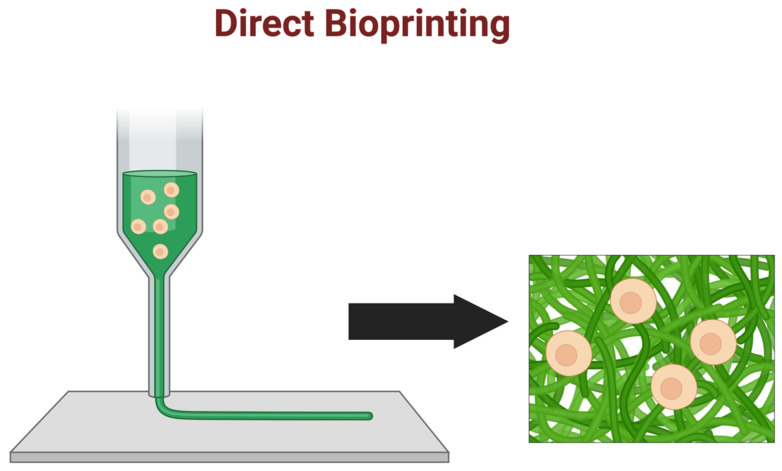
Direct bioprinting: Direct bioprinting methods involve the extrusion or photo-gelation of bioink with cells directly embedded during printing. Post-processing may still be necessary.

Direct bioprinting involves using a printing nozzle to deposit bioink-containing cells (see Figure 5) directly onto a substrate layer-by-layer [76]. This approach provides precise control over the placement and spatial distribution of cells and can create complex tissue structures with high resolution. The ability to precisely control the placement of cells also allows for the creation of microenvironments that promote cell-cell interactions, leading to the formation of functional tissue structures [76]. Direct bioprinting has been used to create various tissue structures, including skin, cartilage, bone, and liver tissues [76]. More recently, direct 3D-bioprinting with human induced pluripotent stem cell (hiPSC)-derived cardiomyocytes embedded in collagen–hyaluronic acid ink has been used to generate functional ring- and ventricle-shaped cardiac tissues [140]. This 3D bioprinting method has also been used to fabricate multi-layered brain tissue by depositing parallel sheets of hPSC-derived cortical neural progenitor cells and hPSC-derived astrocyte suspended in a bioink, based on fibrin gel mixed with hyaluronic acid, to promote the formation of a functional neuronal network in printed tissue [141].

### 5.2. Indirect Bioprinting

**Figure 6:**
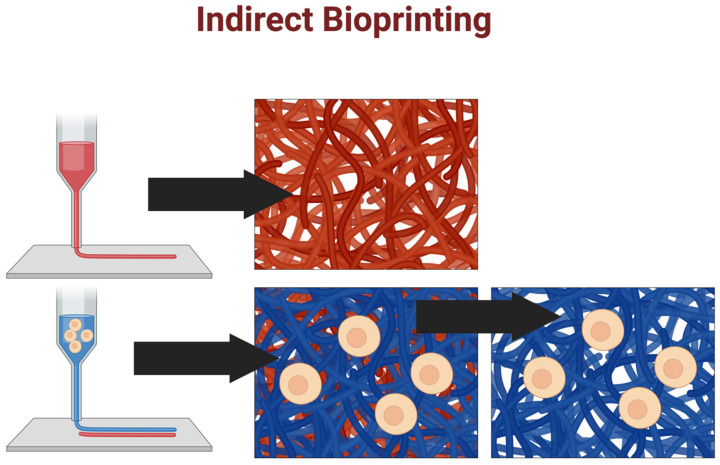
Indirect bioprinting: Indirect bioprinting methods involve printing cell-free materials (shown in red) to support cell-laden material (shown in blue). The support material is then removed to produce the final construct.

Indirect bioprinting involves the creation of a sacrificial framework as a temporary template for the subsequent deposition/fabrication of a polymer scaffold that can incorporate bioactive materials and cells [142]. In this approach, a support material is deposited using a 3D printer and then coated with a thin layer of bioink-containing cells [76]. The support material is then dissolved or removed, leaving behind the printed tissue structure (see Figure 6) [143].

This approach allows for a wider range of cell types, including neuronal cells, which remains challenging [141].

Indirect bioprinting also allows for control over both the external and internal structure, thus allowing the fabrication of scaffolds with advanced architecture, such as vascularized tissue [144], as well as larger tissue structures that may be difficult to print directly due to the limitations of the printing nozzle size. However, this approach can also be challenging, as removing the support material can damage the printed tissue structure, and the sacrificial material may leave residues that can affect cell viability and function.

One study by Lee et al. demonstrated the feasibility of bioprinting a functional BBB model using a combination of endothelial cells and astrocytes [108]. The researchers used a custom-built bioprinting system to deposit alternating endothelial cells and astrocyte layers onto a hydrogel substrate [30]. The BBB model showed enhanced barrier function, as evidenced by increased TEER and decreased permeability to small molecules.

Another study by Campisi et al. used a similar approach to bioprint a 3D BBB model composed of brain microvascular endothelial cells (BMECs) and pericytes [29]. Using a droplet-based bioprinting system, they created a scaffold-free structure cultured under dynamic conditions to promote the formation of tight junctions and other BBB-specific features. The resulting BBB model showed improved barrier function and expression of BBB-specific markers compared to traditional 2D cultures.

Despite the significant progress made in bioprinting BBB models, several challenges must be addressed to translate this technology into clinical applications. These include the development of bioinks with appropriate mechanical and biological properties, the optimization of printing parameters, and the scaling-up of the technology for large-scale tissue fabrication. Addressing these challenges will require interdisciplinary collaboration between biologists, engineers, and clinicians and developing novel approaches and tools to overcome current limitations. Ultimately, the success of bioprinting as a technology will depend on various factors, including the development of new biomaterials, improvements in printing technology, and an increased understanding of the biological processes involved in tissue formation and regeneration.

## 6. Conclusions

The in vitro study of physiological and pathological neurovascular processes thus far continues to demand the use of complementary systems. In this respect, bioassembly technologies integrating multicellular culture environments with controlled perfusion of the vascular systems will provide the tools for creating more physiologically relevant in vitro models that better recapitulate the anatomical and functional features of the NVU and facilitate the study of still elusive neurological disorders as well as the development and testing of novel and more effective CNS drugs. Neurovascular models play a crucial role in drug development, allowing for testing of therapeutic interventions targeting both vascular and central nervous components. Models like the 3D-engineered NVU can help simulate pathological neuroinflammatory and vascular conditions such as stroke [40] and BBB impairments [145,146,147], brain tumors [85], modeling neurodegenerative diseases [148] including Tau pathology in Alzheimer’s Disease [49,146,149], neuronal projection defects in Huntington’s disease [44,53], and Parkinson’s Disease pathogenesis [54,141]. At the same time, these in vitro technologies can also help evaluate the safety and efficacy of treatments for these disorders [41,42,52,53,146,147].

Advanced 3D bioprinting techniques coupled with the development of new bioinks that better mimic the natural extracellular matrix, supporting long-term cell viability and functionality, offer the potential for creating in vitro NVU models that combine the structural brain tissue complexities afforded by organoids systems, with the perfusability of microfluidic platforms while providing the precision, scalability, and reproducibility necessary for large scale pharmacological studies. The development of novel bioinks also continues, with improvements in printability, biocompatibility, and reproducibility, attempting to produce an “ideal” bioink. However, there is still much work to be carried out in this field, and we point the reader towards a very recent comprehensive review on the subject by Gogoi and colleagues [150]. Great strides are being made, but the gap remains significant, with substantial advancements in the physical printing processes (bioprinter technologies) and material science (bioink development) still required to make these technologies more clinically relevant and viable at scale. On the microfluidic front, many currently available brain-on-chip platforms allow for the co-culturing of multiple cell types to promote the development of a more physiologically relevant BBB/NVU with functional vascular channels [40]. These platforms at large provide a stable and fully controllable multiomics system with good reproducibility and the ability to monitor both functional and morphological changes in the BBB/NVU in response to the experimental stimuli, including confocal/fluorescent microscopy [34] (to assess the formation of a continuous vascular endothelial monolayer, the formation, distribution, and oligomerization of tight endothelial junctions between adjacent cells, etc.), and transendothelial electrical resistance measurement for the indirect assessment of BBB integrity [146]. None of the clinically relevant imaging systems, such as Positron Electron Tomography or Magnetic Resonance Imaging, are currently compatible with these platforms (in addition to a lack of compatibility, other hindering factors such as cost-effectiveness, imaging resolution, wide availability outside a clinical setting, and many others need to be considered). Indeed, it is important to remember that, despite the current technological advancements in the field of in vitro tissue/organ modeling, these platforms remain companion research and testing tools for in vivo studies. However, they have acquired much more translational significance through using human cells (primary or iPSC-derived), thus morphing into humanized research and development tools, which could further evolve into patient-specific models for precision medicine research and treatment testing.

## Figures and Tables

**Table 1:** Bioink materials and their properties [139].

Bioink Material	Material Type/Source	Advantages	Disadvantages
Agarose	A polysaccharide extracted from seaweed.	Non-toxic; cross-links readily; highly stable.	Not easily degradable; poor cell adhesion.
Alginate	A biopolymer derived from algae.	Cross-links quickly; highly biocompatible.	Slow to degrade; poor cell adhesion.
Chitosan	A polysaccharide derived from shellfish or fungus.	Highly biocompatible; antibacterial properties.	Slow to gel; pH-sensitive.
Collagen	A protein derived from animal skin or connective tissue.	Highly biologically relevant; highly biocompatible.	pH-sensitive; acid-soluble.
Fibrin	A protein derived from blood.	Highly biologically relevant; highly biocompatible; rapid gelation.	Limited printability.
Elastin	A protein derived from collagen hydrolysis.	High biocompatibility; high water solubility; thermally reversible gelation.	Poor shape fidelity; limited printability.
Graphene	Carbon material, synthesized.	Flexible; electrically conductive.	Low biological relevance.
Hyaluronic acid	Non-sulfated glycosaminoglycan derived from connective tissue.	Fast gelation; promotes cell proliferation	Poor stability.
PCL, PLA, and PLGA	Biodegradable thermoplastic polymer/copolymer.	High strength; rigidity; excellent printability.	Low cell adhesion and proliferation.
Pluronic	Copolymer.	Shear-thinning material; printable at room temperatures.	Does not support long-term cell culture

## Data Availability

Not applicable.
